# Meaningful conversations in living with and treating chronic conditions: development of the ICAN discussion aid

**DOI:** 10.1186/s12913-016-1742-6

**Published:** 2016-09-23

**Authors:** Kasey R. Boehmer, Ian G. Hargraves, Summer V. Allen, Marc R. Matthews, Christina Maher, Victor M. Montori

**Affiliations:** 1 Knowledge and Evaluation Research (KER) Unit, Mayo Clinic, 200 First Street SW, Rochester, MN 55905 USA; 2 Department of Family Medicine, Mayo Clinic, 200 First Street SW, Rochester, MN 55905 USA

**Keywords:** Multiple chronic conditions, Communication, Minimally disruptive medicine

## Abstract

**Background:**

The needs of the growing population of complex patients with multiple chronic conditions calls for a different approach to care. Clinical teams need to acknowledge, respect, and support the work that patients do and the capacity they mobilize to enact this work, and to adapt and self-manage. Tools that enable this approach to care are needed.

**Methods:**

Using user-centered design principles, we set out to create a discussion aid for use by patients, clinicians, and other health professionals during clinical encounters. We observed clinical encounters, visited patient homes, and dialogued with patient support groups. We then developed and tested prototypes in routine clinical practice. Then we refined a final prototype with extensive stakeholder feedback.

**Results:**

From this process resulted the ICAN Discussion Aid, a tool completed by the patient and reviewed during the consultation in which patients classified domains that contribute to capacity as sources of burden or satisfaction; clinical demands were also classified as sources of help or burden. The clinical review facilitated by ICAN generates hypotheses regarding why some treatment plans may be problematic and may not be enacted in the patient’s situation.

**Conclusion:**

We successfully created a discussion aid to elucidate and share insights about the capacity patients have to enact the treatment plan and hypotheses as to why this plan may or may not be enacted. Next steps involve the evaluation of the impact of the ICAN Discussion Aid on clinical encounters with a variety of health professionals and the impact of ICAN-informed treatment plans on patient-important outcomes.

## Background

In part due to the successes of public health and health care and to the ageing of the population, the incidence of multiple chronic conditions (MCC) is increasing: 3 in 4 Americans 65 and older live with MCC, and the prevalence is increasing also in younger people [1, 2]. In an era in which adults are more likely to live with chronic conditions than not, health can no longer be defined as the absence of disease. Instead, a new definition has been proposed: the ability to adapt and to self-manage [3]. The epidemic of MCCs and this new definition require a dramatic shift in the culture of providing care: when health care cannot cure, it must instead understand and develop the capacity of patients to adapt and self-manage to the chronic conditions that almost inevitably will enter their lives.

Minimally Disruptive Medicine (MDM) and its underpinning conceptual model, the Cumulative Complexity Model provide practitioners with useful heuristics to approach this shift [4, 5]. First, there is significant, underappreciated, patient work necessary to implement care plans, particularly as conditions and demands accumulate [6, 7]. Attending appointments, taking medications, shopping for and preparing healthy food, enacting an active lifestyle, dealing with administrative tasks related to insurance, scheduling appointments, refilling prescriptions, and self-monitoring are activities that take time, effort, sense-making, and attention [8, 9]. This patient work must be enacted along with the work required to fulfill roles in life. Second, patients must invest *capacity* - effort, time, emotion, help, and attention - to implement life and patient work. This capacity may be reduced by their illness from symptoms of fatigue, pain, or general functional decline; it may also be reduced by lack of money or stable housing, and other forms of scarcity [5]. With enough capacity to face the extant workload patients can access and use healthcare and enact self-care, which has favorable downstream consequences on health outcomes [5]. If workload exceeds capacity, the opposite can be true. Indeed, when health care is unable to note that poor outcomes are a consequence of the imbalance of workload and capacity, treatment may be intensified, worsening the situation [5].

The Burden of Treatment Theory further illuminates patients’ capacity to adapt and self-manage by illustrating that it is not simply the presence or absence of resources. Rather, it proposes that capacity is critically dependent on the social settings in which it operates, hinging on the patient’s social skill and social network [10]. The concepts of workload, capacity, and treatment burden, are complex, interdependent, and *subjective*. Because of this, measuring patient capacity or treatment burden will only have partial value to clinicians and healthcare professionals who seek to partner with their patients to understand their situation and consider ways to improve it. Yet, the brevity and crowding of tasks typical of clinic visits offer little to these patients beyond technical solutions to specific complaints. Although dynamic, clinicians must gain an understanding of the patient capacity at this time. The difficulty of this task to both patients and clinicians is well documented [11–15]. A partnership between patients and clinicians appears necessary to successfully care for patients with MCC [4, 16, 17]. This partnership requires a different conversation, accomplished in the same brief and crowded encounters designed historically to address the technical, acute problems of health. Tools and techniques that enable the effective and efficient conversations necessary to assess patient capacity and to use this understanding to advance plans that are more likely to be implemented and have impact are needed.

Therefore, the aim of this study was to develop a discussion aid for use during the encounter between patients, clinicians, and other health professionals to understand patient capacity, workload, and treatment burden.

## Methods

The development process for the ICAN Discussion Aid took place in clinical practice at Mayo Clinic and various community locations in Olmsted County, Minnesota. All clinicians involved consented to observation of their clinical encounters, and, where applicable, to the use of a design prototype during the encounter. Patients were eligible for inclusion in the study if they had at least one chronic condition. While Minimally Disruptive Medicine primarily focuses on patients with multiple chronic conditions, it seeks to recognize complexity in life that often complicates healthcare plans. For this reason, we did not exclude patients with only one chronic condition to capture patients who might have life complexity complicating care even with a single condition. Patients with cognitive impairments or any other barrier to informed consent were excluded from participation. All patients that were observed during clinical encounters, home visits, and support groups consented to participation in the study. The Mayo Clinic Institutional Review Board approved the study procedures.

**Fig. 1:**
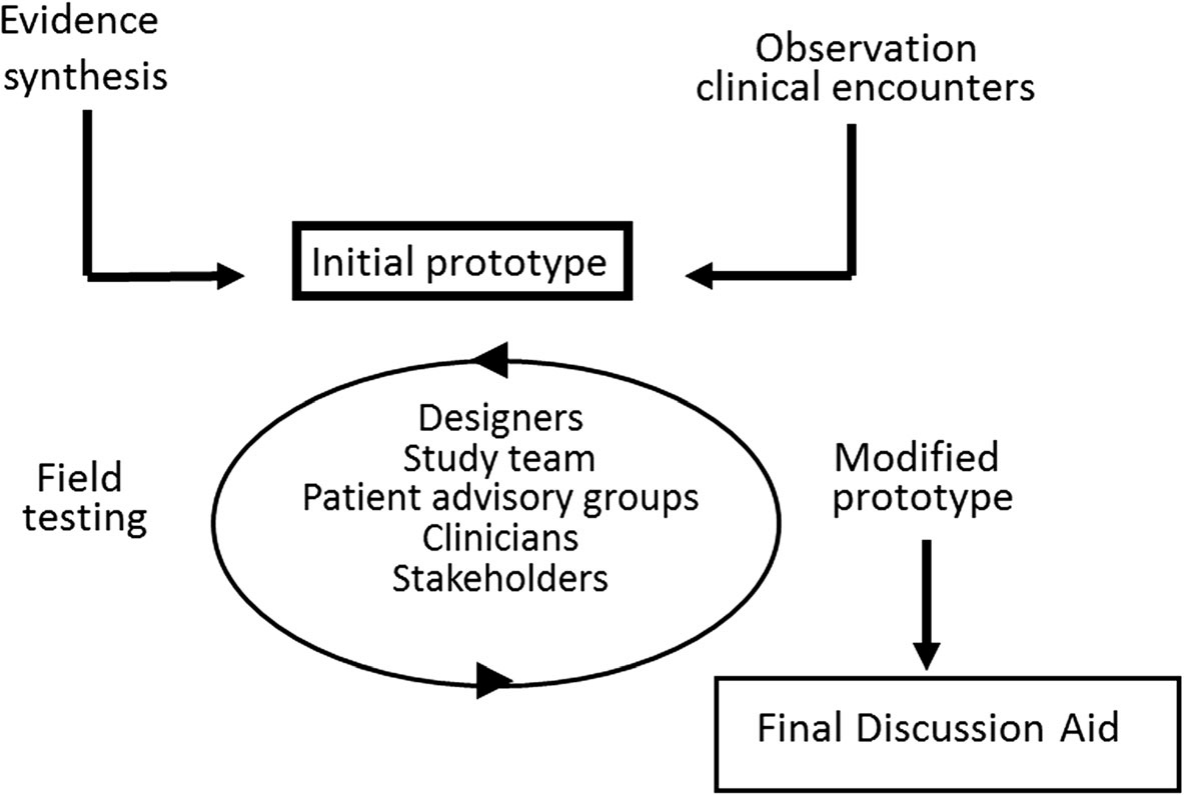
Discussion Aid Development Process

We used a two-part development process, illustrated in Fig. 1. One component, reported elsewhere, was a systematic review of 110 qualitative studies reporting insights that bear on the concept of patient capacity. Briefly, that review highlighted the importance of patient Biography, Resources, Environment, accomplishing patient and life Work, and Social Functioning (BREWS), for advancing patients’ capacity [18]. The second component, the focus of this report, was the user-centered design of a discussion aid about patient capacity. We have used this design approach in the development of decision aids for use during clinical consultations [19, 20].

The design process took place in four phases, and began with direct observations (phase I). These included observations of 1) clinical encounters in primary care (*n* = 15), aimed at understanding if and how matters related to patient capacity were discussed in clinical care; and 2) observations of nurse home visits with patients (*n* = 5), aimed at understanding if and how matters of patient capacity were discussed in the patients’ home. Because of the pertinent setting, we assumed conversations in patient homes would be richer than similar ones in the clinic. We also observed two patient support groups to understand how patients talked about their capacity to handle the work of chronic disease with their peers. At the conclusion of these observations, we debriefed and reviewed our findings with our patient advisory group, ten community members with chronic conditions [21].

Based on our observations in phase I, we generated three prototypes. We hypothesized that each one, when used in conversations, could draw out patient capacity. To evaluate these, we partnered with two primary care clinicians (SVA, MRM), who were selected based on a long-standing interest in Minimally Disruptive Medicine and willingness to alter their clinical routines during prototyping. We observed two to three clinical encounters with each prototype per clinician during this phase (phase II, *n* = 15). Phase II informed new hypotheses and three new prototypes, building on parts of the conversation that worked well and abandoning those that did not. These prototypes were used by the same two clinicians in subsequent encounters (phase III, *n* = 13).

During phase III, one prototype showed promise for illuminating the dynamic interplay of patient capacity and treatment burden, and we sought to further refine it (phase IV, *n* = 5). After three additional iterations with this prototype, we were satisfied with the 9th prototype, deeming it the final version. We conducted observations of the 8th and 9th prototype in use, for which we recruited two additional primary care clinicians and four specialty care clinicians (phase IV, *n* = 13). Additionally, we conducted observations with the 9th prototype in the practice of a group of wellness coaches seeing patients with chronic conditions to explore the feasibility and pertinence of its use by other health professionals (phase IV, *n* = 13).

## Results

### Phase I

Characteristics of patients in all clinical encounter observations that informed design are shown in Table 1. Patients ranged in ages from 18 to 87, with a mean age of 51.3 and had an average of 4.6 chronic conditions (range 1–11). It is important to also note that in addition to medical complexity, 32.4 % of these patients had known non-medical complexity, such as family distress, financial problems, housing issues, or complex substance abuse histories. We did not seek to target specific conditions and a wide variety were represented including, but not limited to: diabetes, COPD, depression, anxiety, hypertension, heart failure, fibromyalgia, and other chronic pain conditions.

**Table 1:** Patient Demographic Characteristics

Patient demographics clinical encounters (*n* = 74)	
Age, mean (SD)	51.3 (18.0)
Number of Conditions, mean (SD)	4.6 (2.5)
Male	23.0 %
Non-Medical Complexity (i.e., family distress, housing problems, complex substance abuse history)	32.4 %
Race/Ethnicity	
White/Not Hispanic	93.2 %
White/Hispanic	2.7 %
Other Hispanic Origin	1.4 %
African American	1.4 %
Unknown	1.4 %

Four key findings resulted from Phase I observations in clinical encounters. First, information about patients’ capacity and context sometimes arose naturally, but haphazardly throughout the visit. Second, patients exhibited learned behaviors in relation to the medical encounter. Patients who spoke freely and enjoyed talking about their life upon meeting them, changed completely during the encounter. There, they spoke only of their medical conditions and immediate concerns, and none of the life issues they had mentioned to us previously. This is in part in response to clinicians’ choice of opening questions. For example, “what can I do for you today?” yielded clinical requests. Since clinicians may be less likely to be able to “do” something about the patient’s life challenges, these simply did not arise. Fourth, assessments that offered insights about the patient’s capacity were not used for this purpose. For example, in one encounter, the patient had filled out multiple questionnaires, one regarding depression, one regarding anxiety, and one regarding alcohol consumption. Despite being available during the encounter, these were never reviewed or discussed.

During the home visit observations, we met patients who were the recipients of resources from the healthcare or public health system (e.g., home health services, housekeeping services, physical aids). Despite these resources in support of each patient’s capacity, not all patients were doing well. The noticeable difference between home visits in which nurses and patients appeared more successful conversationally and those that did not was the effort the nurse made in extracting what gave the person’s life meaning, what made the patient tick, or what made them get out of bed each morning despite the challenges. The receipt of additional resources delivered without clear connection to the patient’s situation did not appear to mitigate patients’ struggles to self-manage.

Finally, we presented the idea to our patient advisory group as exploring how to have conversations about their situation with clinicians so as to better align the treatment plans developed to the circumstances of each patient’s life and to patient ability to enact these plans. At first, participants struggled with this concept; it was clearly foreign to them, even after years of living with chronic conditions. Midway, a patient boldly interjected: *“You mean the doctor might ask me how I am coping with my disease? Well, that would change the world.”* The discussion that followed highlighted the importance of patients’ ability to adapt and self-manage so that illness and health care not disrupt the life they found value in living.

### Phase II

**Fig. 2:**
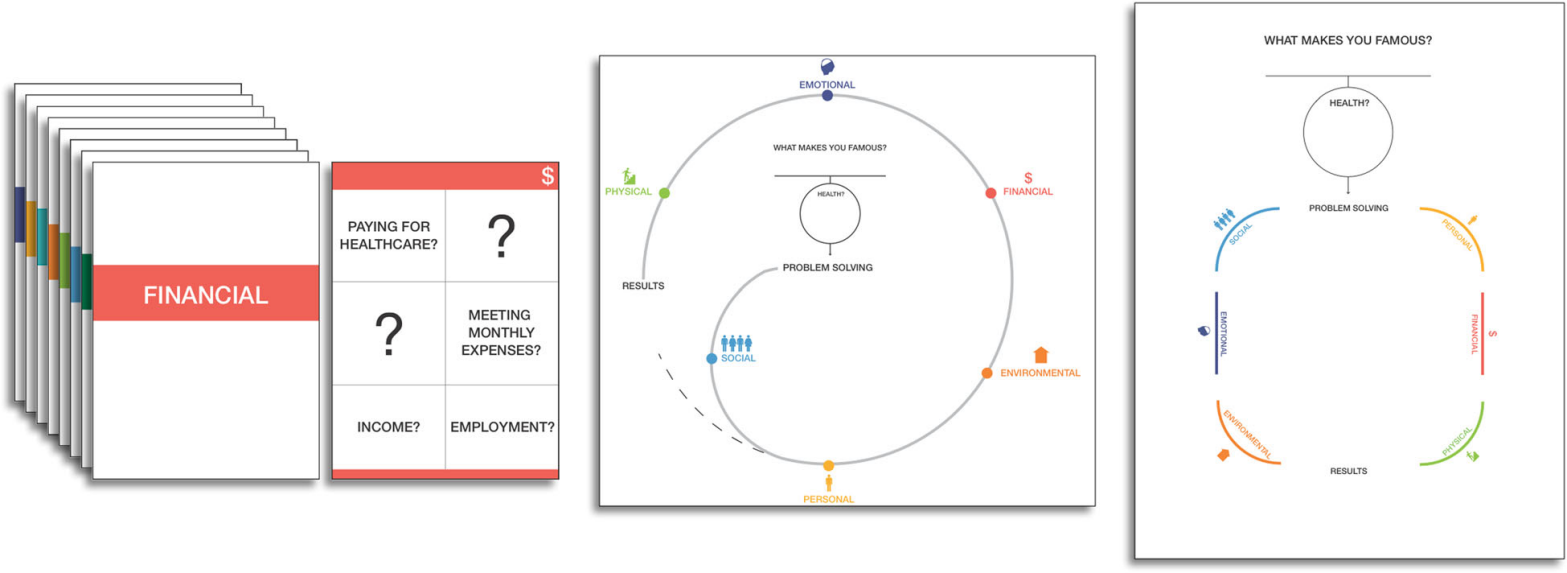
Phase II Prototypes

These key findings informed the development of our prototypes. The three prototypes that we developed included a deck of cards the patient could hold and two different paper diagrams that clinicians could fill as they had a conversation with the patient (Fig. 2). The prototypes: 1) were embedded in the beginning of the clinical encounter, to prevent information about patient context emerging haphazardly, disjointedly, or too late to inform the treatment plan; 2) used more open-ended opening questions; 3) brought out contextual information beyond resources (e.g., finances, instrumental support, etc.); and 4) did not quantify patients’ capacity, but rather elicited this information through dialogue between patients and clinicians.

In Phase II, we observed that the prototypes showed the most promise when they were shared between patient and clinician. In other words, there needed to be more than a clinician checklist. The words that we used to describe patient capacity (Physical, Emotional, Personal, Financial, Social, and Environmental) were friendlier to researchers and physicians than to patients. To fully allow patients to participate, these needed to be reconsidered. The opening question “What makes you famous?” had the intended effect of allowing life information to be permissible in the conversation, and in some cases brought out fun and insightful information. In other instances, it felt awkward and warranted reconsideration.

### Phase III

**Fig. 3:**
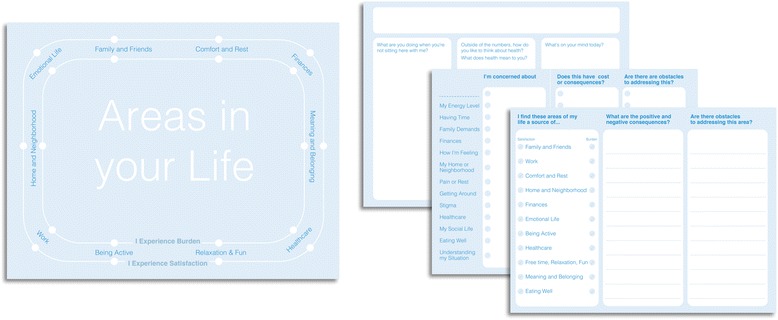
Phase III Prototypes

The three new prototypes contained the original four design principles, but offered three opening questions instead of one, each serving a specific purpose, and used language friendlier to patients. The prototypes included a clinician question sheet, and three different patient-filled sheets. Each patient-filled sheet was tried separately, in combination with the clinician questions (Fig. 3). This round of prototyping sought to contrast two different ways of discussing patient capacity. The first one elicited patient concerns. The second invited patients to indicate whether each area of their life was a source of satisfaction/help, a burden, or both. This latter approach seemed more effective in creating the conversations we intended to promote than the focus on specific concerns. When discussing concerns, conversation often fell flat. Patients either felt uncomfortable discussing them, or didn’t perceive things as “concerns,” and didn’t bring them up. The design underwent refinement in phase IV, building on the dynamic tradeoffs patients needed to make in their lives.

### Phase IV

When discussing areas of their life, we observed that patients discussed tradeoffs or competing priorities. For example, a patient mentioned that she and her husband lived in an elderly community cooperative. While this offered distinct socialization and safety benefits at their age, there were many fellow dwellers that used wheelchairs or canes. Since they did not require physical aids, this was sometimes depressing and made the patient think if that would be their future as well. We had explicitly asked about consequences of areas of their life and obstacles to overcome as a result, but such prompting was mostly unnecessary. We hypothesized that patients were also making similar tradeoffs in the healthcare work they had been asked to do, either among competing healthcare tasks or competing life priorities. Therefore, we modified the prototype to remove additional life prompts. We also inserted a column to ask patients about the things that they had been asked to do to care for their health. At first, we populated this list for the patient. However, we quickly moved to a version that patients could populate for themselves and learned they were skillful in stating what they had been asked to do.

Our final modifications to the prototype included a change from a color version to black and white, and to reorder the list of items in the “life” column. We learned from stakeholder feedback, that older patients struggled with the color contrast and preferred a highly contrasted, black-and-white version. Additionally, our stakeholders indicated that it might be useful to group the list by what clinicians generally handle versus issues that call for other team members to take part in the care of the patient. The first group, from My Family and Friends to Faith and Personal Meaning, identifies important contextual factors that clinicians recognize, if nothing to else to enroll social workers, community health workers, wellness coaches, and others in addressing these. The second group, from Being Active to Eating Well, represents contextual factors on which clinicians could intervene directly.

We observed clinicians and health professionals share the patient-filled portion of the discussion aid in multiple ways. Some began by asking about a burden, some about a satisfaction. However, the questions, “what stands out to you?” or “tell me a little bit about what you’ve filled here” appeared to spark the most conversation.

### Finalizing

**Fig. 4:**
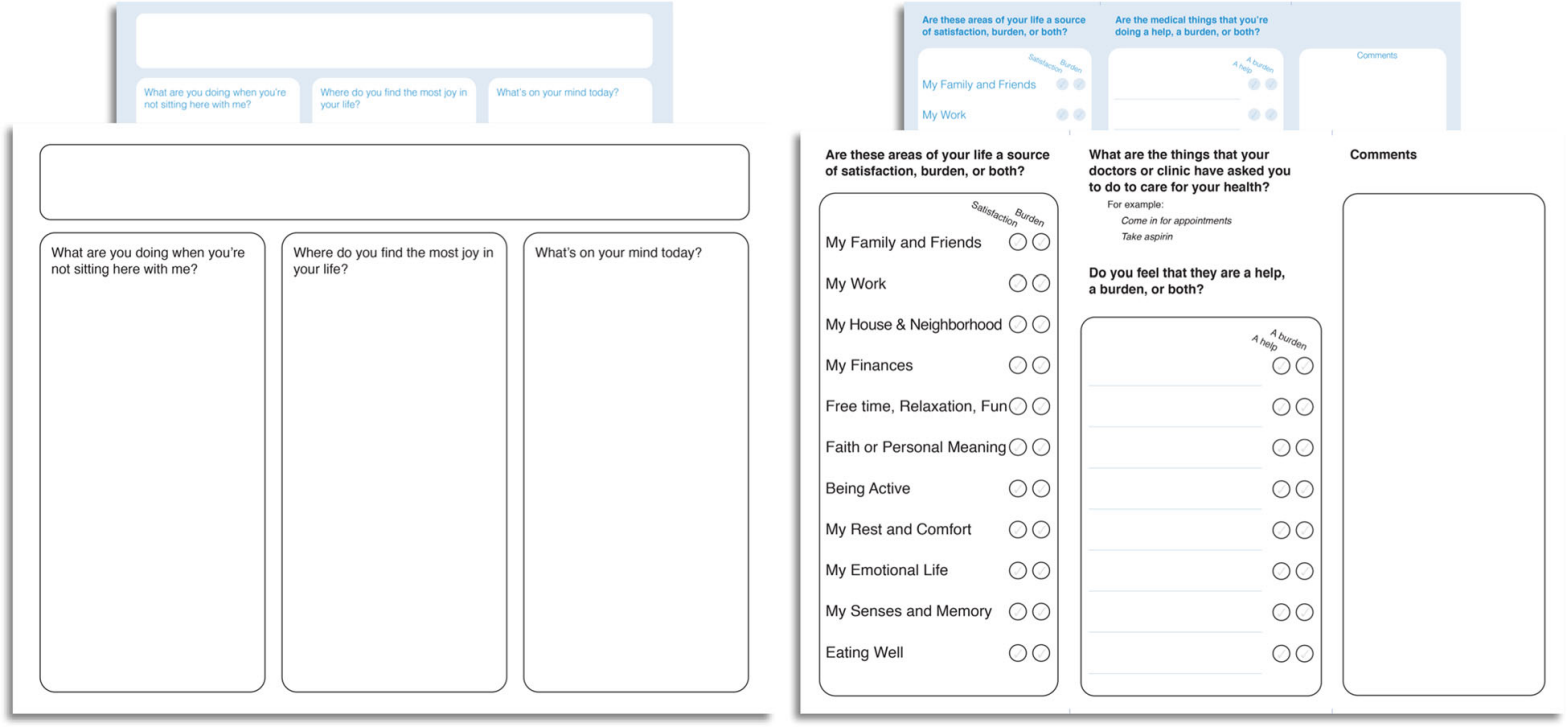
Final ICAN Discussion Aid

**Fig. 5:**
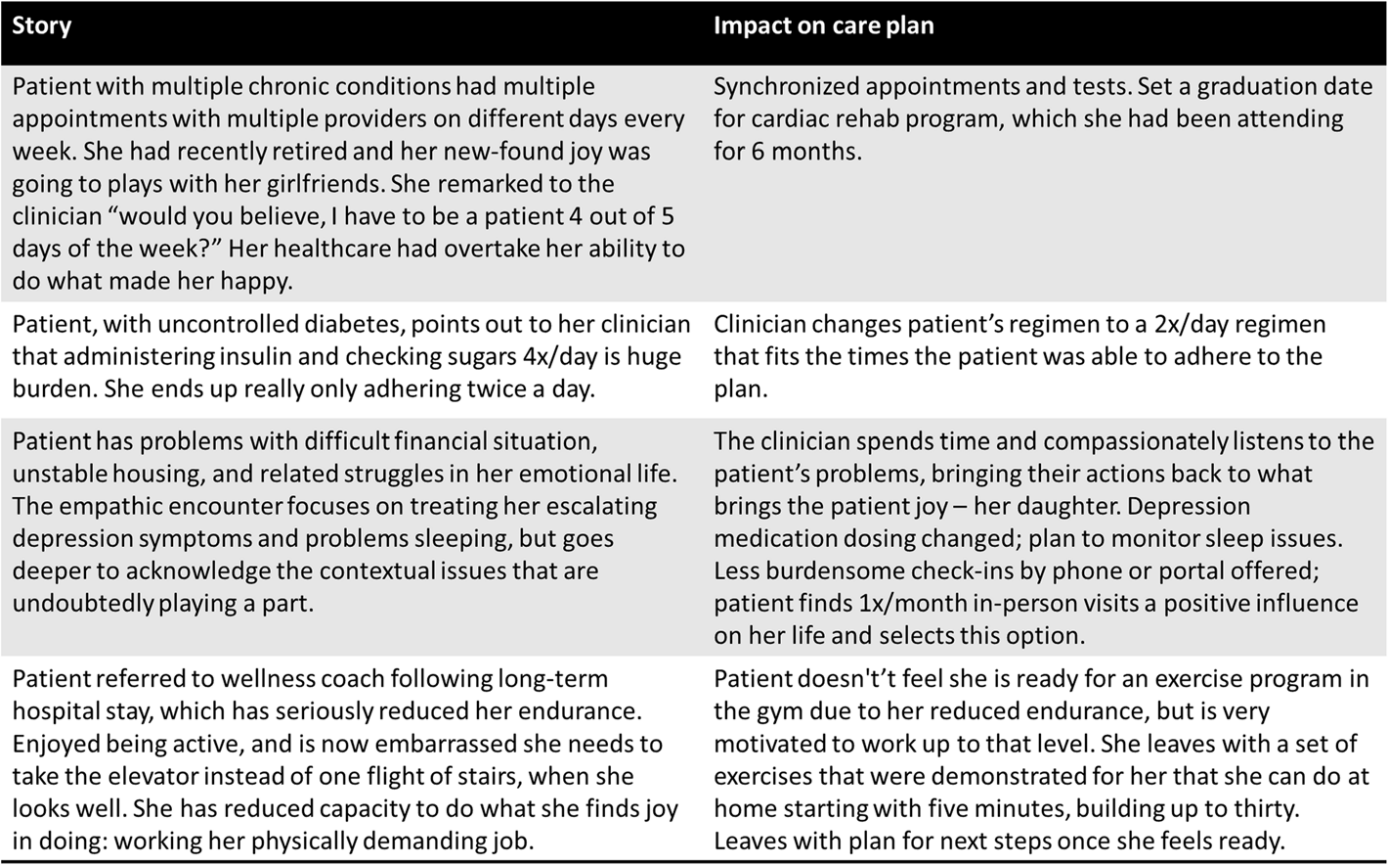
Encounter Highlights

We deemed the ICAN Discussion Aid (Fig. 4) finished when we began to notice distinct changes in the patient-clinician conversation from our initial observations. Conversations using the final prototype brought up contextual issues of patient capacity and treatment burden early, highlighting the competing priorities in life and healthcare, and in many cases resulting in changes to treatment plans (Fig. 5). Furthermore, when we observed the prototype’s use with other health professionals, it did not require any modifications, demonstrating its versatility.

Briefly, the final version of the ICAN discussion aid is used in the following way: while waiting for the clinician, the patient takes ~3 min to fill ICAN. The clinician asks one or more of three opening questions, which are used in conversation but not depicted to the patient: 1) “What are you doing when you’re not sitting here with me?” brings forth information about typical day-to-day and competing priorities, important for formulating treatment fit. 2) “Where do you find the most joy?” allows clinicians to assess if the patient is struggling with biographical disruption from their treatment and illness [22–24], as well as ensure that treatment ties to or does not overwhelm the patient’s meaning-making activities (family or community life, work, hobbies), and 3) “What’s on your mind today?” focuses on the visit today, but without unnecessarily narrowing the options to healthcare concerns. The clinician then explores what the patient filled by asking “what stands out to you on this sheet you filled?” The leftmost patient-filled column is designed for maximum clinical efficiency and team coordination. The first group of items, from “My Family and Friends” to “Faith and Personal Meaning,” identifies coping sources. If serious problems exist in these areas, clinicians may want to enroll social workers, community health workers, health coaches, and others to address them. The second group, from “Being Active” to “Eating Well,” represents health issues on which clinicians’ can intervene directly. When used by other healthcare professionals, they may be able to intervene directly on the first group of items, whereas they may need to reach out to the patient’s clinician to discuss the second group. Finally, the second column of ICAN elicits the demands healthcare places on patients’ lives, which can also be explored through dialogue.

## Discussion

### Summary of study

We set out to develop a Discussion Aid for use in clinical encounters by patients and clinicians to assess patient’s capacity to handle the work of being a patient in the context of their lives. Through a rigorous, user-centered design process, we have created the ICAN Discussion Aid. This aid demonstrates early evidence of success in illuminating how patients’ lives and health care interact. ICAN can be used to inform longitudinal relationships between patients and their healthcare team across a broad range of healthcare professionals.

### Limitations and strengths

The user-centered design process that informed the development of the aid is a considerable strength of this work. Rather than informing our design through formal focus groups or in the conference rooms of researchers and clinicians, we took our design directly to the practice. This was purposeful to ensure that the final ICAN Discussion Aid would be novel, practical, useful, and friendly to clinicians, health professionals, and their patients. Another strength of ICAN’s design is its ability to open up a range of hypotheses for patients and their healthcare team to explore together. For example, for new patients, clinicians can quickly get to know their patient, what might work in their context, and why certain treatment plans might be problematic. This approach surfaces concerns immediately, but also establishes a firm foundation for future interaction. In ongoing patient-clinician relationships, where communication or progress is stalled, ICAN may uncover hypotheses for why this is the case. This strength is notable when compared to previous assessments of function. For example, Huber suggests measuring health as functioning using The COOP/Wonca Functional Health Assessment Charts. These present six different dimensions of health scored 1 to 5, each supported by drawings [3, 25]. These assessments quantify some elements of capacity, which may not tell the full story, and when practitioners must cumulatively assess many domains at one time, can become increasingly cumbersome in the clinical encounter. Furthermore, our observations suggest that quantifying capacity may not necessarily engage two parties in a discussion about the results.

These strengths are not without limitations. For example, we cannot conclude that ICAN will be useful in all situations or for all clinicians, or that its use will improve patient outcomes, all outside the bounds of this stage of our project. Future research will need to study the impact of ICAN on the clinical encounter, the patient’s relationship with their clinicians and healthcare teams, their burden of treatment and illness, and on patient-centered health outcomes.

### Implications for practice

The ICAN Discussion Aid is the first discussion aid that can be implemented into practice in pursuit of Minimally Disruptive Medicine [4]. Specifically, it provides an avenue for clinicians to implement three of the four principles of MDM. First, it illuminates the necessary information to establish the burden of treatment offering clinicians hypotheses to answer, “what is the most effective least burdensome treatment for this person now?” Consider three otherwise similar patients with uncontrolled type 2 diabetes. By using ICAN, a clinician learns that one has a strong social network and travels extensively for work; that the other is a farmer who spends time out in the fields and worrying about his fluctuating income; and that the other is retired and spends time enjoying her home, but with almost no social support. The most effective, least burdensome treatment program to improve diabetes control in each of these patients will likely be different.

Second, ICAN can be used to encourage coordination in clinical practice. Future research should seek to understand how health systems can organize care teams around this new-found understanding of each patient’s capacity, which is more meaningful than the current social history found in electronic health records. Third, and likely most importantly, it allows patients and their healthcare team to prioritize care from the patient perspective. ICAN underscores what the patient values doing and being in life, i.e., what does their normal day look like and where do they find joy, and provides the building blocks of a conversation that can prioritize care around how health care and life can work in synergy to support that perspective.

Ultimately, the opening of these possibilities establishes meaningful relationships with patients and their healthcare team, and may lead to treatment plans that are more likely to fit within the patient’s life. Based on the conceptual underpinnings of Minimally Disruptive Medicine and related theories and frameworks, these changes should positively impact the patient’s ability to access and use healthcare and enact self-care, and ultimately experience favorable health outcomes [5]. We hope that attending to patient’s capacity can also help eliminate contextual errors in care [26]. Furthermore, ICAN’s implementation should improve health care’s own capacity to cope with the task of organizing and delivering care to more complex patients including those with MCC and helping them achieve the ability to adapt and self-manage in the face of chronic disease.

## Conclusion

Through a rigorous user-centered design process, we developed the ICAN Discussion Aid, which should support clinical conversations that acknowledge, respect, and support the underappreciated work of being a patient and patients’ capacity to carry out that work. These conversations should support longitudinal relationships with patients and their healthcare teams that further patients’ ability to adapt and self-manage while pursing meaningful lives. ICAN deserves future testing to determine its efficacy in promoting Minimally Disruptive Medicine.

## Abbreviations

MCC: Multiple chronic conditions
MDM: Minimally Disruptive Medicine
